# Supplementary material for: Lipid nanoparticle-encapsulated mRNA therapy corrects serum total bilirubin level in Crigler-Najjar syndrome mouse model
Source: Mol Ther Methods Clin Dev. 2023 Feb 15;29:32–9. doi: 10.1016/j.omtm.2023.02.007 (PMC10017950; doi:10.1016/j.omtm.2023.02.007)
Supplement: Document S1. Figures S1–S3 [file mmc1.pdf]

OMTM, Volume *29*

Supplemental information

Lipid nanoparticle-encapsulated mRNA therapy corrects serum total bilirubin level in Crigler-Najjar syndrome mouse model

Jenny A. Greig, Joanna K. Chorazeczewski, Vivek Chowdhary, Melanie K. Smith, Matthew Jennis, James C. Tarrant, Elizabeth L. Buza, Kimberly Coughlan, Paolo G.V. Martini, and James M. Wilson

Supplemental Data

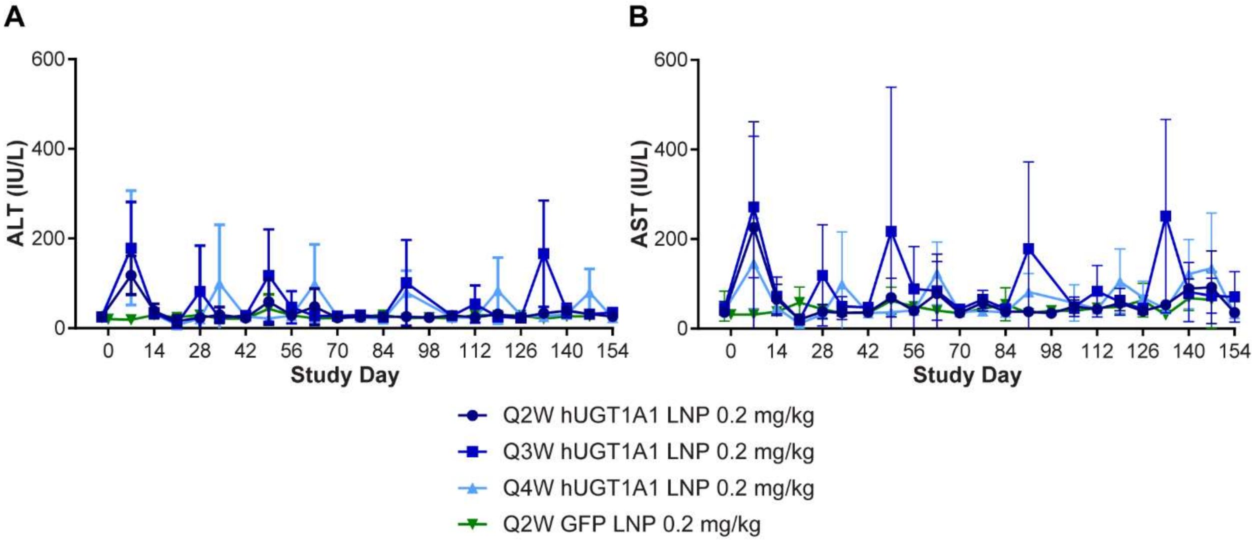

Figure S1. Liver transaminases following repeated administration of LNP encapsulated mRNA therapy in the *Ugt1* KO mouse.
Adult *Ugt1* KO mice (n=5/group) were injected IV with multiple administrations of 0.2 mg/kg LNP-encapsulated *hUGT1A1* mRNA (A) every two weeks (Q2W), (B) every three weeks (Q3W), and (D) every four weeks (Q4W). An additional group of mice received IV injections of 0.2 mg/kg LNP encapsulated GFP mRNA Q2W as a control. Blood was collected at selected time points to evaluate ALT (A) and AST (B). Values are presented as mean ± SD.

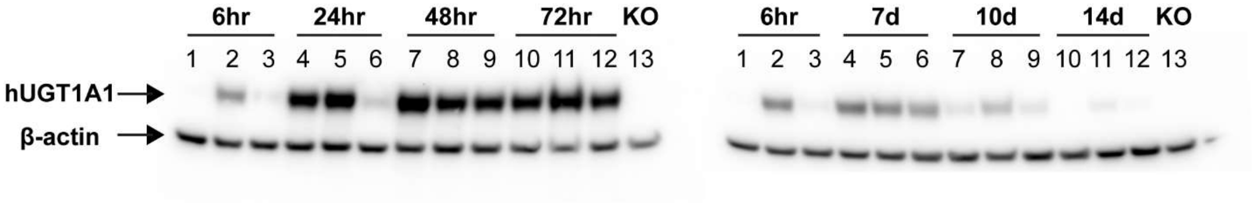

Figure S2. Evaluation of *hUGT1A1* protein pharmacokinetics following IV administration of LNP encapsulated *hUGT1A1* mRNA in C57BL/6J mice.
Adult C57BL/6J mice (n=3/group) were injected by IV with 0.5 mg/kg of LNP encapsulated *hUGT1A1* mRNA and sacrificed at the indicated time points. Livers were harvested for Western blot analysis to determine *hUGT1A1* protein expression.

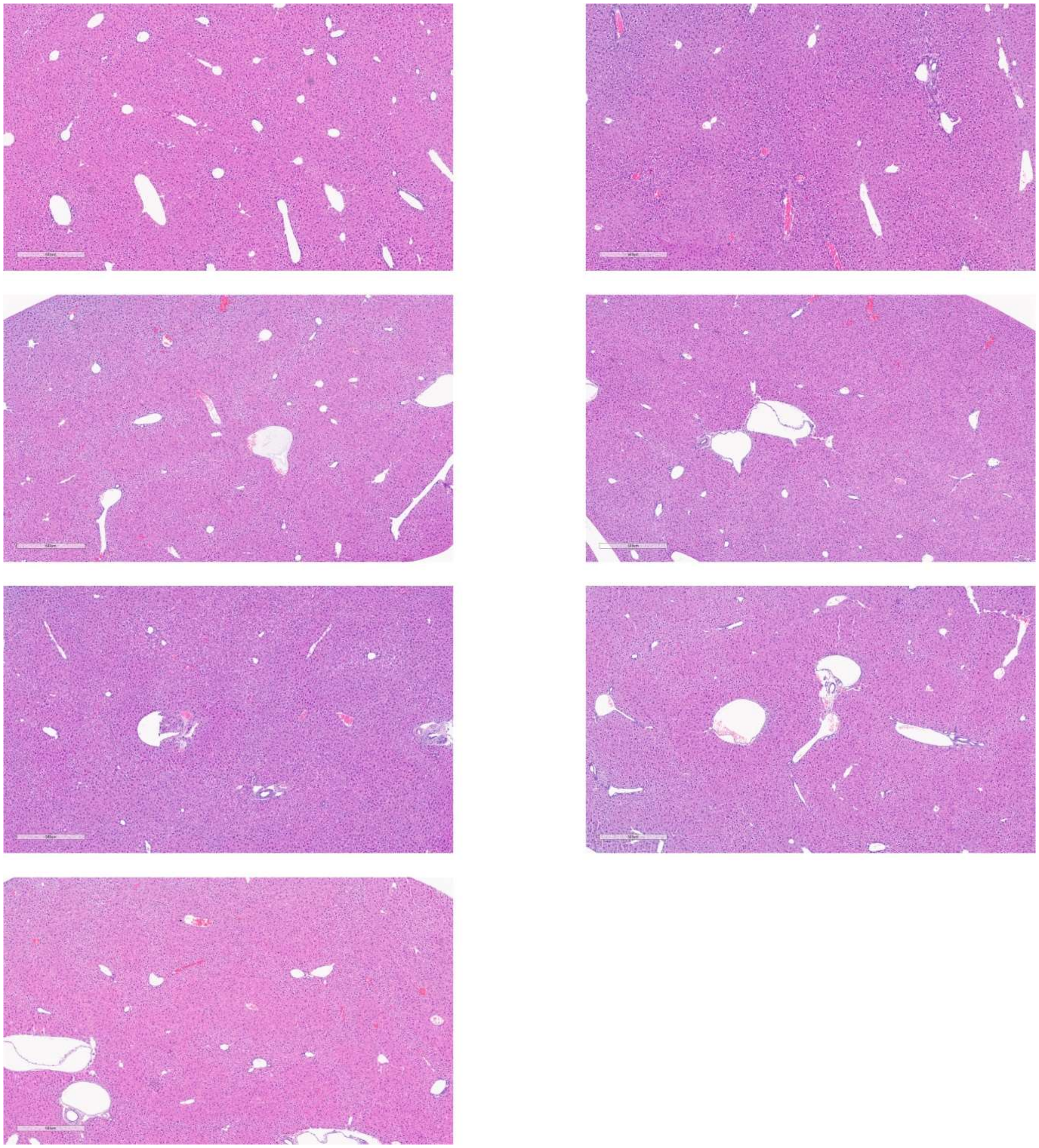

Figure S3. Images of hematoxylin and eosin (H&E)–stained liver sections of *Ugt1* KO mice. Newborn *Ugt1* KO mice were administered 0.5 mg/kg of LNP-encapsulated hUGT1A1 mRNA via a temporal vein, and tissues were harvested >100 days post-administration. Scale bar 500μm.
