# Supplementary material for: Lipid nanoparticle-encapsulated mRNA therapy corrects serum total bilirubin level in Crigler-Najjar syndrome mouse model
Source: Mol Ther Methods Clin Dev. 2023 Feb 15;29:32–9. doi: 10.1016/j.omtm.2023.02.007 (PMC10017950; doi:10.1016/j.omtm.2023.02.007)
Supplement: Document S2. Article plus supplemental information [file mmc2.pdf]

Molecular Therapy
Methods & Clinical Development
Original Article

Lipid nanoparticle-encapsulated mRNA therapy corrects serum total bilirubin level in Crigler-Najjar syndrome mouse model

Jenny A. Greig,[1] Joanna K. Chorazeczewski,[1] Vivek Chowdhary,[1] Melanie K. Smith,[1] Matthew Jennis,[1] James C. Tarrant,[1] Elizabeth L. Buza,[1] Kimberly Coughlan,[2] Paolo G.V. Martini,[2] and James M. Wilson[1]

[1]Gene Therapy Program, Department of Medicine, University of Pennsylvania, Philadelphia, PA, USA; [2]Moderna, Inc., Cambridge, MA, USA

Crigler-Najjar syndrome is a rare disorder of bilirubin metabolism caused by uridine diphosphate glucuronosyl transferase 1A1 (*UGT1A1*) mutations characterized by hyperbilirubinemia and jaundice. No cure currently exists; treatment options are limited to phototherapy, whose effectiveness diminishes over time, and liver transplantation. Here, we evaluated the therapeutic potential of systemically administered, lipid nanoparticle-encapsulated human *UGT1A1* (h*UGT1A1*) mRNA therapy in a Crigler-Najjar mouse model. *Ugt1* knockout mice were rescued from lethal post-natal hyperbilirubinemia by phototherapy. These adult *Ugt1* knockout mice were then administered a single lipid nanoparticle-encapsulated h*UGT1A1* mRNA dose. Within 24 h, serum total bilirubin levels decreased from 15 mg/dL (256 μmol/L) to <0.5 mg/dL (9 μmol/L), i.e., slightly above wild-type levels. This reduction was sustained for 2 weeks before bilirubin levels rose and returned to pretreatment levels by day 42 post-administration. Sustained reductions in total bilirubin levels were achieved by repeated administration of the mRNA product in a frequency-dependent manner. We were also able to rescue the neonatal lethality phenotype seen in *Ugt1* knockout mice with a single lipid nanoparticle dose, which suggests that this may be a treatment modality appropriate for metabolic crisis situations. Therefore, lipid nanoparticle-encapsulated h*UGT1A1* mRNA may represent a potential treatment for Crigler-Najjar syndrome.

INTRODUCTION

Crigler-Najjar syndrome is an ultra-rare disorder of bilirubin metabolism caused by mutations in the uridine diphosphate glucuronosyl transferase 1A1 (*UGT1A1*) gene.[1-5] The most severe form of Crigler-Najjar syndrome is called type 1 (CN1) and presents as persistent jaundice at or soon after birth as a result of hyperbilirubinemia >20 mg/dL (342 μmol/L).[6] The lack of functional UGT1A1 prevents bilirubin from being conjugated in hepatocytes prior to excretion,[7] and the transit of these abnormally high levels of bilirubin across the blood-brain barrier can cause kernicterus, which can result in irreversible neurological damage.

Currently, no cure exists for CN1. Clinical management primarily consists of phototherapy, which negatively affects the quality of life, is variable in the duration required per patient, is only partially effective at lowering bilirubin levels, and becomes less effective over time.[8,9] Other illnesses can also lead to severe bilirubin excursions in which multiple clinical strategies are deployed to reverse the crisis and prevent kernicterus. Liver transplantation is a treatment option, although there is potential for morbidity and, in rare cases, mortality associated with the surgical procedure and long-term immunosuppression. These limited treatment options highlight the tremendous unmet clinical need and the potential positive impact of a novel therapeutic approach.

Previous attempts at alternative treatment strategies have utilized adeno-associated virus vectors to achieve therapeutic expression of human *UGT1A1* (h*UGT1A1*).[10-18] While these gene therapy approaches have proven efficacious in other liver diseases, they are limited by interfering antibodies and concerns over the durability of expression. Others have evaluated the therapeutic potential of a lipid nanoparticle (LNP)-encapsulated mRNA therapy for the treatment of CN1.[19] mRNA delivery by LNPs results in hepatocyte distribution through opsonization, followed by receptor-mediated endocytosis.[20-25] By delivering mRNA to hepatocytes (i.e., the site of action for UGT1A1), this approach can potentially treat the fundamental cause of CN1. Previous studies utilized the naturally occurring Gunn rat model to evaluate LNP-encapsulated mRNA therapy for CN1.[19] Gunn rats lack active hepatic UGT1A1 and exhibit sustained hyperbilirubinemia due to a frameshift mutation caused by a single base deletion in *UGT1A1*.[26,27]

Here, we evaluated the therapeutic potential of an LNP-encapsulated mRNA therapy following systemic administration in a CN1 mouse model, the *Ugt1* knockout (KO) mouse. Compared with the less severe phenotype displayed in the Gunn rat, the *Ugt1* KO mouse displays hyperbilirubinemia in the immediate post-natal period that is

Received 18 September 2021; accepted 10 February 2023;
https://doi.org/10.1016/j.omtm.2023.02.007.

Correspondence: James M. Wilson, MD, PhD, Gene Therapy Program, Perelman School of Medicine, University of Pennsylvania, 125 South 31st Street, Suite 1200, Philadelphia, PA 19104, USA.
E-mail: wilsonjm@upenn.edu

This is an open access article under the CC BY-NC-ND license (http://creativecommons.org/licenses/by-nc-nd/4.0/).

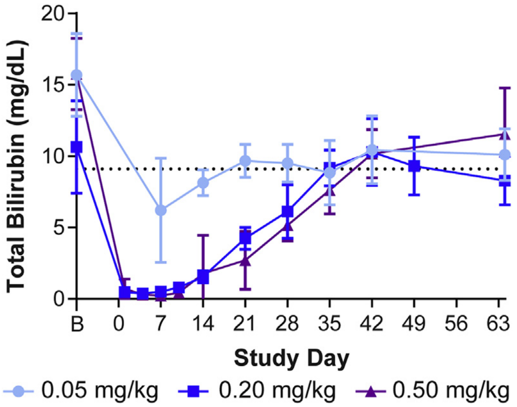

Figure 1. Single dose of LNP-encapsulated mRNA therapy normalizes serum bilirubin levels in the *Ugt1* KO mouse

Adult *Ugt1* KO mice were injected i.v. with a single administration of LNP-encapsulated h*UGT1A1* mRNA at a dose of 0.05 (n = 3), 0.2 (n = 9), or 0.5 mg/kg (n = 9). Blood was collected for n = 3 mice per dose at selected time points for evaluation of serum total bilirubin levels. The dotted line indicates average serum total bilirubin levels in untreated adult *Ugt1* KO mice (9.1 ± 3 mg/dL).[11] B, baseline bleeds performed between day −7 and day −3 prior to treatment. Values presented as mean ± SD.

fatal within the first week of life without intervention.[14,28] Intervention with phototherapy (i.e., exposure to blue fluorescent light for 12 h/day for up to 21 days after birth) rescues this lethal neonatal phenotype and allows affected mice to survive to adulthood, albeit while displaying the hyperbilirubinemia characteristic of the disease (i.e., elevated serum total bilirubin levels of 9.1 ± 3 mg/dL following weaning from phototherapy).[11,14,29] We evaluated the pharmacokinetics and efficacy of repeated systemic dosing of LNP-encapsulated h*UGT1A1* mRNA in adult *Ugt1* KO mice and investigated whether the neonatal lethality phenotype seen in *Ugt1* KO mice could be rescued with a single LNP dose.

RESULTS

A single dose of LNP-encapsulated mRNA therapy normalizes serum bilirubin in the *Ugt1* KO mouse

To evaluate the ability of LNP-encapsulated mRNA to treat CN1, we utilized the *Ugt1* KO mouse model. In order to ameliorate the hyperbilirubinemia phenotype characteristic of this model, a proof-of-principle study was performed whereby phototherapy-rescued adult *Ugt1* KO mice were systemically administered a single dose of LNP-encapsulated h*UGT1A1* mRNA. Mice were injected with a single dose of 0.05, 0.2, or 0.5 mg/kg of LNP-encapsulated mRNA via the tail vein (Figure 1). Mice dosed with 0.2 or 0.5 mg/kg exhibited reductions in serum total bilirubin levels to approximately wild-type levels (0.4–0.5 mg/dL) that were sustained for 2 weeks, at which time bilirubin levels began to rise before returning to baseline levels of hyperbilirubinemia by day 42 post-administration. There was no significant difference between serum total bilirubin levels in mice dosed with either 0.2 or 0.5 mg/kg, with both doses significantly improving the phenotype compared to mice treated with 0.05 mg/kg LNP-encapsulated h*UGT1A1* mRNA.

Repeated administration of LNP-encapsulated mRNA therapy sustains reductions in serum bilirubin levels in the *Ugt1* KO mouse

Following completion of the washout period after the single dose of LNP-encapsulated mRNA (Figure 1), the same *Ugt1* KO mice were repeatedly administered LNP-encapsulated h*UGT1A1* mRNA. By varying the dosing interval, we determined the optimal dosing regimen to achieve sustained reductions in serum total bilirubin levels. Mice were treated every 2 weeks (Q2W; Figure 2A), every 3 weeks (Q3W; Figure 2B), or every 4 weeks (Q4W; Figure 2C) with 0.2 mg/kg LNP-encapsulated h*UGT1A1* mRNA or Q2W with a negative control LNP containing green fluorescent protein (*GFP*) mRNA (same control group presented in Figures 2A–2C).

Q2W administration of the GFP LNP did not alter serum total bilirubin levels across the study duration, and the control *Ugt1* KO mice displayed the typical degree of variability in baseline levels (8.3 ± 2.1 mg/dL; Figure 2). Mice treated with Q2W administration of the LNP h*UGT1A1* mRNA had a sustained reduction in serum total bilirubin levels for the duration of the study, with some fluctuations occurring later in the study (Figure 2). Over the course of the study, it became more challenging to perform repeated intravenous (i.v.) administrations in these mice due to limited access sites, which may have resulted in incomplete dosing. Dosing Q3W or Q4W resulted in reductions in serum bilirubin levels for 7–14 days post-injection (similar to that seen following a single dose [Figure 1]), which returned to baseline hyperbilirubinemia levels prior to the next dose 21 or 28 days later, respectively (Figures 2B and 2C). While liver transaminase elevations were present directly following LNP administration across the groups, we did not observe any sustained elevations throughout the study duration (Figure S1). The livers were harvested at necropsy, and we performed histopathological analyses (Table 1). Minimal-to-mild findings of mononuclear cell infiltration were seen across all groups, including mice that received the GFP LNP. Other minimal findings of single hepatocyte necrosis, pigmented macrophages, Kupffer cell infiltrates, and oval cell hyperplasia were also observed in some mice but were considered incidental.

While all dosing intervals significantly reduced serum total bilirubin levels compared with control *Ugt1* KO mice, Q2W dosing achieved average levels of 2.78 mg/dL, which was a substantial improvement on levels following Q3W and Q4W dosing. The duration of action of the LNP h*UGT1A1* mRNA is dependent on both mRNA and protein stability; our data indicate that a biweekly dosing regimen can effectively and sustainably reduce bilirubin levels in the *Ugt1* KO mouse.

Time course of LNP-encapsulated mRNA therapy for CN

To determine the time course of both the h*UGT1A1* mRNA and expressed protein *in vivo,* adult C57BL/6J mice were injected with 0.5 mg/kg LNP-encapsulated h*UGT1A1* mRNA and sacrificed at 6 h, 24 h, 48 h, 72 h, 7 days, 10 days, and 14 days post-administration. Necropsies were performed, and livers were harvested for immunohistological analyses (Figure 3A) to evaluate RNA levels by *in situ*

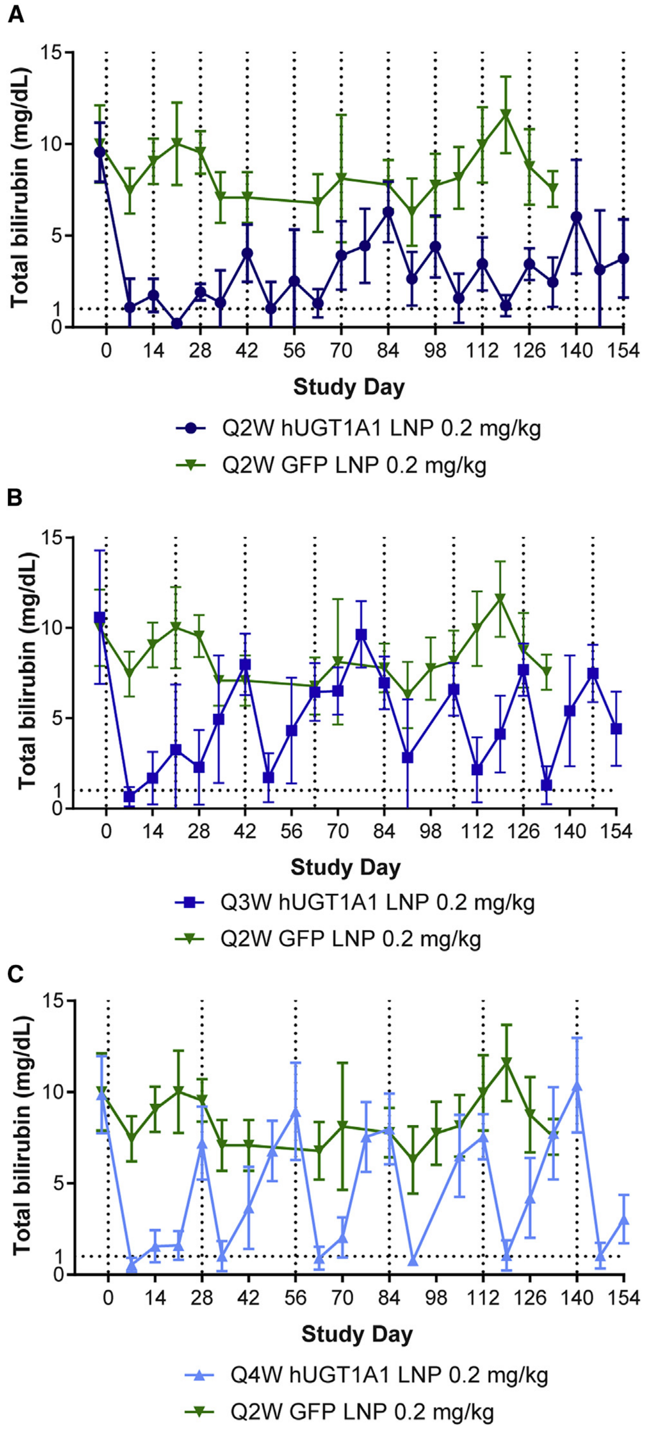

Figure 2. Repeated administration of LNP-encapsulated mRNA therapy sustains reductions in serum bilirubin levels in the *Ugt1* KO mouse

Adult *Ugt1* KO mice (n = 5/group) were injected i.v. with multiple administrations of 0.2 mg/kg LNP-encapsulated h*UGT1A1* mRNA (A) every two weeks (Q2W), (B) every three weeks (Q3W), or (C) every four weeks (Q4W). Dotted lines indicate the day of dosing. An additional group of mice received i.v. injections of 0.2 mg/kg LNP-encapsulated GFP mRNA Q2W as a control (green line in A, B, and C). Blood was collected at selected time points for evaluation of serum total bilirubin levels, including prior to dosing. Values presented as mean ± SD.

hybridization (ISH) (Figure 3B) and hUGT1A1 protein levels by Western blot (Figures 3C and S2). There was detectable RNA in the liver up to 72 h post-LNP administration by ISH, and h*UGT1A1* expression from the delivered mRNA was sustained through 10 days post-LNP administration (Figure 3).

Optimization of the h*UGT1A1* mRNA sequence in adult *Ugt1* KO mice

We further developed the h*UGT1A1* mRNA sequence and evaluated the efficacy of this second mRNA with a different 5′ untranslated region (UTR) with the same Cap, open reading frame (ORF), 3′ UTR, and tail background compared with the original mRNA in a repeat of the single-dose study described above. Adult *Ugt1* KO mice were dosed at 0.2 mg/kg LNP-encapsulated h*UGT1A1* mRNA (either original or redesigned sequence) in a single i.v. injection. An additional group of mice received the vehicle as a control (phosphate-buffered saline [PBS]). Serum total bilirubin levels were decreased in a similar manner in the original and redesigned mRNA treatment groups, although a lower level was achieved on day 7 with the redesigned mRNA sequence (to 1.4 mg/dL or 15% of baseline levels compared with 2.2 mg/dL or 30% of baseline with the original mRNA) (Figure 4A).

A single administration of LNP-encapsulated mRNA therapy to newborn *Ugt1* KO mice rescues neonatal lethality

We evaluated the utility of this LNP-encapsulated mRNA therapy with the redesigned sequence to rescue *Ugt1* KO mice from their lethal neonatal phenotype in which they require euthanasia within the first week of life.[11,28] Litters of mice resulting from heterozygous $Ugt1^{+/-}$ mating were injected with 0.1, 0.2, or 0.5 mg/kg LNP-encapsulated mRNA therapy or vehicle on the first day of life (Figure 4B). Mice were followed for survival; vehicle-treated *Ugt1* KO mice died by day 3, mimicking what we observed in untreated *Ugt1* KO mice.[11] Treatment with 0.1 mg/kg or 0.2 mg/kg of the redesigned LNP-encapsulated h*UGT1A1* mRNA sequence enhanced survival to a maximum of 17 and 19 days post-birth, respectively (Figure 4B). A single treatment with the highest dose (0.5 mg/kg) allowed all *Ugt1* KO mice to survive until weaning (days 21–28); this group demonstrated survival to >100 days, which is the latest we observed the animals and is well beyond lethal neonatal exposure. While the transient nature of LNP-encapsulated mRNA therapy was sufficient to rescue the neonatal *Ugt1* KO mice, these animals had hyperbilirubinemia levels comparable to phototherapy-rescued *Ugt1* KO animals at day 28. Liver samples collected at necropsy (>100 days post-administration) were evaluated for histopathology; minimal findings of infiltrates were seen in two out of seven *Ugt1* KO mice administered with the highest dose and were considered to be background findings (Figure S3).

Table 1. Liver histopathological analysis following repeated administration of LNP-encapsulated mRNA therapy in the *Ugt1* KO mouse

Incidence/severity table				
Tissue	No. animals affected/total treated			
Treatment	h*UGT1A1* Q2W	h*UGT1A1* Q3W	h*UGT1A1* Q4W	*GFP* Q2W
Liver				
Necrosis, single hepatocyte				
Grade 1	0/4	1/5	0/5	0/5
Mononuclear cell infiltrate				
Grade 1	4/4	3/5	2/5	3/5
Grade 2	0/4	2/5	3/5	2/5
Pigmented macrophage and Kupffer cell infiltrate				
Grade 1	0/4	1/5	4/5	0/5
Oval cell hyperplasia				
Grade 1	1/4	1/5	0/5	0/5

Adult *Ugt1* KO mice (n = 5/group) were i.v. injected with multiple administrations of 0.2 mg/kg LNP-encapsulated human uridine diphosphate glucuronosyl transferase 1A1 (h*UGT1A1*) mRNA every 2 weeks (Q2W), every 3 weeks (Q3W), or every 4 weeks (Q4W). An additional group of mice received i.v. injections of 0.2 mg/kg LNP-encapsulated green fluorescent protein (GFP) mRNA Q2W as a control. Livers were harvested at necropsy, and histopathological analysis was performed.

DISCUSSION

We have shown the efficacy of LNP-encapsulated mRNA for treating hyperbilirubinemia resulting from UGT1A1 deficiency in the *Ugt1* KO mouse model of CN1. After establishing the pharmacokinetics of both the treatment and the response following a single dose of LNP in adult mice, we were able to determine that mRNA dosing every two weeks (Q2W) was required to prevent the peaks in serum total bilirubin levels indicative of a reversal to hyperbilirubinemia that were seen in the other groups dosed less frequently. Therefore, repeated administration of LNP-encapsulated h*UGT1A1* mRNA may hold potential for both short- and long-term treatment of CN1. These data are similar to those generated previously in the Gunn rat; however, we were able to demonstrate the efficacy of this approach in an animal model with an enhanced severity phenotype (the *Ugt1* KO mouse) compared with that of the Gunn rat,[19] suggesting that this approach could be effective upon translation to patients with CN1 in the clinic.

In mice repeatedly administered with LNP-encapsulated h*UGT1A1* mRNA, we observed some fluctuations in serum total bilirubin levels later in the study, which correlated with it becoming more challenging to perform repeated i.v. administrations in these mice. The resulting fluctuations may then have resulted from incomplete dosing. An alternative explanation could be a potential immune response to the h*UGT1A1* protein in the animal model of CN1.[13,16,18,30] While we did not evaluate adaptive immune responses to hUGT1A1 protein or cells expressing this protein, mice treated with biweekly administration of the h*UGT1A1* mRNA retained sustained reductions in serum total bilirubin levels for the duration of the study (154 days), suggesting an absence of deleterious antibodies or T cells.[31] In this study, we also observed transient liver transaminase elevations directly following LNP administration, similar to those previously reported following delivery of LNP-mRNA treatment for methylmalonic acidemia.[32] In addition, histopathological changes in the liver were limited to minimal-to-mild mononuclear cell infiltrations across all groups at necropsy following up to 12 administrations with LNP h*UGT1A1* mRNA with no evidence of hepatocyte injury.

Current clinical management of patients with CN1 consists predominantly of daily phototherapy to stimulate the photoisomerization of bilirubin into water-soluble isomers. The number of hours of phototherapy required per day can range from >10 h to maintain young patients with CN1 below the threshold for encephalopathy[8,33] to 20 h per day.[34] Unfortunately, phototherapy is only partially effective at lowering bilirubin levels, and patients with CN1 have lifelong hyperbilirubinemia (16 ± 5 mg/dL)[8] and an ongoing risk of reaching threshold levels for kernicterus during times of illness or stress. This highly burdensome treatment approach also becomes less effective as the patient ages due to alterations in body surface area, thickening of the skin, and decreased compliance due to its obvious inconvenience.[35] While recurrent i.v. administration of an LNP-encapsulated mRNA therapy at less than monthly intervals would be associated with some inconveniences, including those based on the dosing interval, infusion volume and time, and the location where dosing could be performed, there is the expectation that this treatment approach would address the cause of CN1 without imposing the same limitations on travel, employment, or lifestyle as phototherapy.

In addition to evaluating LNP-encapsulated *hUGT1A1* mRNA as a long-term treatment approach following repeated administration, we also evaluated this therapy to treat acute metabolic crises in CN1. When bilirubin levels are being managed by phototherapy, there is still a risk of other adverse events resulting in severe excursions into hyperbilirubinemia. In addition, other illnesses, stressors, or even just the interruption of phototherapy for a short period can result in brain damage. We believe that LNP-encapsulated h*UGT1A1* mRNA could be rapidly deployed to treat acute metabolic crises and prevent kernicterus. We modeled this in the *Ugt1* KO mice by evaluating the efficacy of this mRNA treatment to rescue the neonatal lethality seen within the first week of life in this strain. A single 0.5 mg/kg dose of LNP-encapsulated mRNA within the first 24 h of birth completely rescued the *Ugt1* KO mice. Together with the data from the pharmacokinetic evaluations, this suggests that administration of LNP-encapsulated mRNA at the time of crisis could be sufficient to reverse hyperbilirubinemia within hours.

As hepatocytes are the primary site of gene expression following LNP-encapsulated mRNA therapy,[21-25] there are potential implications for patients with liver function issues. Except for a deficiency in UGT1A1 activity, the structure and function of the liver are generally normal in CN1 patients. However, there have been recent case reports of intrahepatic cholestasis and fibrosis, which increase with age.[9,36] While the effect of liver fibrosis on efficacy and safety of LNP-encapsulated mRNA therapy has not yet been evaluated, the degree of fibrosis reportedly correlates with the lifetime average bilirubin levels in

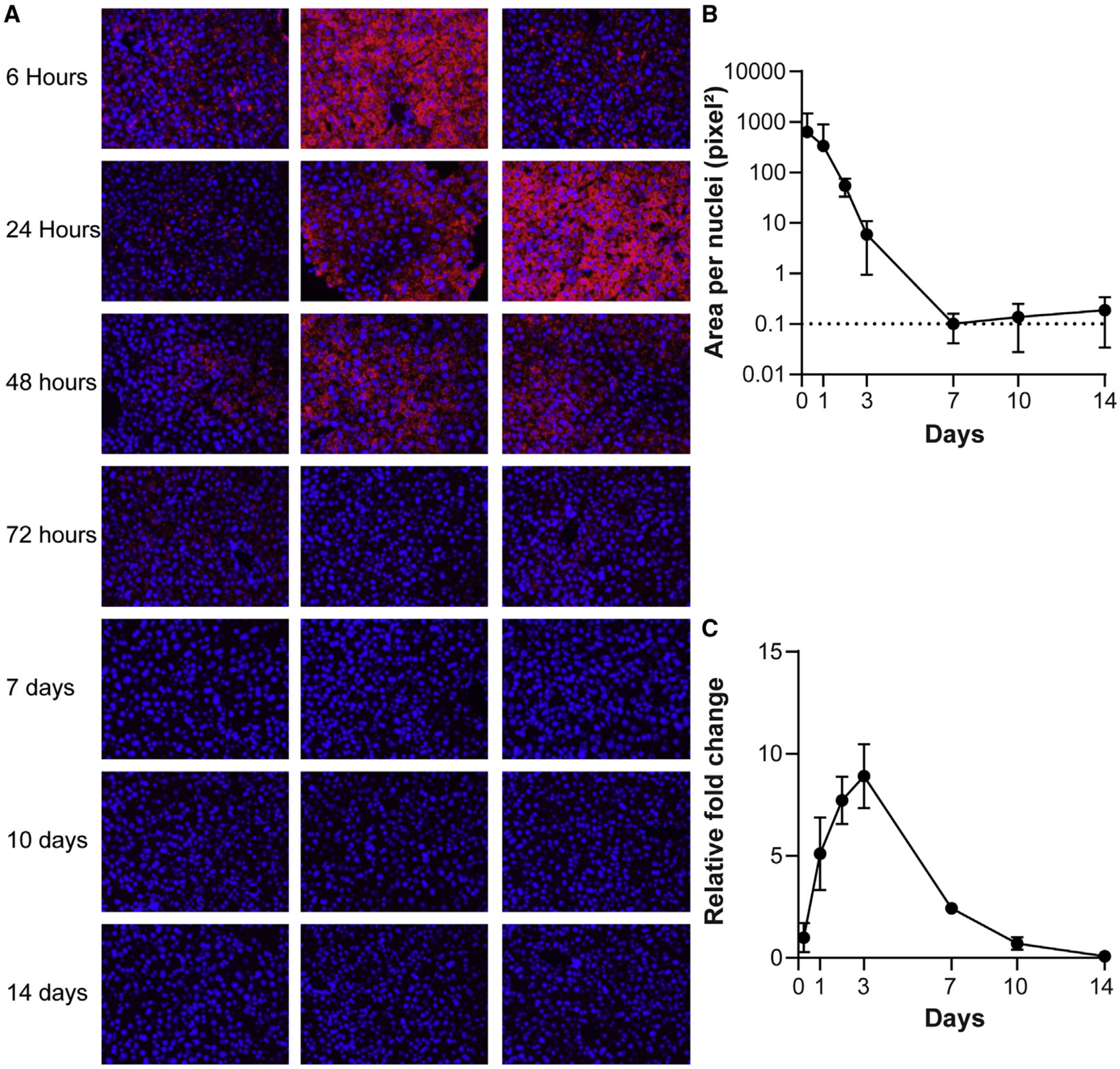

Figure 3. Evaluation of RNA and protein time course following i.v. administration of LNP-encapsulated h*UGT1A1* mRNA in C57BL/6J mice
Adult C57BL/6J mice (n = 3/group) were i.v. injected with 0.5 mg/kg of LNP-encapsulated h*UGT1A1* mRNA and sacrificed at the indicated time points. The liver was harvested for ISH and Western blot analyses. (A) ISH was performed on liver sections with h*UGT1A1* mRNA detected as red staining with DAPI nuclear stain in blue. (B) Images were quantified to determine h*UGT1A1* mRNA-positive area relative to the number of nuclei. (C) Western blots were performed to determine hUGT1A1 protein expression normalized to β-actin. Data are presented as fold change relative to signal strength at 6 h. Values presented as mean ± SD.

patients,[9] suggesting that early intervention with chronic LNP-encapsulated mRNA therapy could prevent future liver fibrosis. We therefore conclude that LNP mRNA therapy may be suitable for both the long-term treatment of CN1 and for managing acute hyperbilirubinemic metabolic crises associated with this disease.

MATERIALS AND METHODS

mRNA/LNP production

A sequence-optimized mRNA encoding hUGT1A1 was synthesized *in vitro* using an optimized T7 RNA polymerase-mediated transcription reaction with complete replacement of N1-methylpseudouridine, as previously described.[37] The reaction included a DNA template containing the protein ORF flanked by 5′ UTR and 3′ UTR sequences and was terminated by an encoded polyA tail. After transcription, the Cap 1 structure was added to the 5′ end using vaccinia capping enzyme (New England Biolabs) and vaccinia 2′ O-methyltransferase (New England Biolabs). The mRNA was purified by oligo-dT affinity purification, buffer exchanged by tangential flow filtration into sodium acetate (pH 5), sterile filtered, and kept frozen at −20°C until further use. A second 5′ UTR (proprietary sequence) in the same Cap, ORF, 3′ UTR, and tail background was also evaluated.

The mRNA was encapsulated in an LNP through a modified ethanol-drop nanoprecipitation process as described previously.[38] In brief,

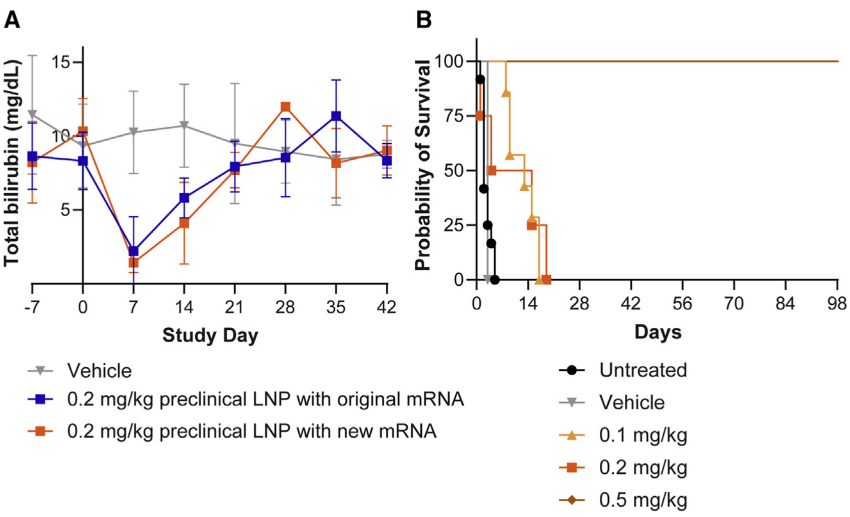

Figure 4. Optimization of the h*UGT1A1* mRNA sequence increases efficacy in adult *Ugt1* KO mice, and a single dose rescues neonatal *Ugt1* KO mice

(A) Adult *Ugt1* KO mice were i.v. injected with a single administration of LNP-encapsulated h*UGT1A1* mRNA (either original sequence, n = 4, or redesigned sequence, n = 3) at a dose of 0.2 mg/kg. An additional group of mice received i.v. injections of vehicle as a control (n = 7). Blood was collected at selected time points for evaluation of serum total bilirubin levels. Values presented as mean ± SD. (B) Newborn *Ugt1* KO mice were i.v. injected with a single administration of vehicle (n = 1) or 0.1 (n = 7), 0.2 (n = 4), or 0.5 mg/kg (n = 7) LNP-encapsulated redesigned mRNA sequence on day 0 of life. Mice were evaluated for survival. Untreated mouse (n = 12) data were previously published.[11]

ionizable, structural, helper, and polyethylene glycol lipids were mixed with mRNA in acetate buffer (pH 5). The mixture was neutralized with Tris-Cl (pH 7.5), sucrose was added as a cryoprotectant, and the final solution was sterile filtered. Vials were filled with formulated LNP and stored frozen at −70°C until further use. The drug product underwent analytical characterization, which included the determination of particle size and polydispersity, encapsulation, mRNA purity, double-stranded RNA content, osmolality, pH, endotoxin, and bioburden, and the material was deemed acceptable for *in vivo* study.

Mice

Breeding pairs of heterozygous *Ugt1*$^{+/-}$ mice (mixed B6 and 129 background strains) were obtained from The Jackson Laboratory (Bar Harbor, ME, USA), and a colony was maintained at the University of Pennsylvania under specific-pathogen-free conditions. All mice used for this study were derived from this colony. All animal procedures and protocols were approved by the Institutional Animal Care and Use Committee of the University of Pennsylvania.

For single and repeated dosing studies in adult *Ugt1* KO mice (13–43 weeks of age), litters of mice were exposed to phototherapy immediately after birth (blue fluorescent light, λ = 450 nm; 10–30 μW/cm^2/nm) for 12 h per day for up to 21 days after birth to generate phototherapy-rescued adult *Ugt1* KO mice. For the single-dose studies, adult *Ugt1* KO mice received an i.v. injection with 0.05, 0.2, or 0.5 mg/kg LNP-encapsulated h*UGT1A1* mRNA diluted in PBS via the tail vein as a single administration. Additional *Ugt1* KO mice received a single i.v. injection with 0.2 mg/kg of either the original or redesigned *hUGT1A1* mRNA sequence or vehicle (20 mM Tris, 8% sucrose buffer [pH 7.4]). Mice were monitored for serum total bilirubin levels throughout the in-life phase of the studies.

For the repeated dosing study, the same adult *Ugt1* KO mice from the single dose group (following completion of the washout period) received multiple i.v. injections of 0.2 mg/kg LNP-encapsulated *hUGT1A1* mRNA Q2W, Q3W, or Q4W, with a cohort of mice receiving 0.2 mg/kg LNP-encapsulated GFP mRNA Q2W as a control. Blood samples were collected weekly by submandibular bleeds and prior to dosing with LNP-encapsulated *hUGT1A1* mRNA. Mice were necropsied at least 28 days after their last LNP administration.

In order to evaluate the time course, male C57BL/6J mice were purchased from The Jackson Laboratory and received an i.v. injection of 0.5 mg/kg LNP-encapsulated *hUGT1A1* mRNA via the tail vein (diluted in PBS). Mice were necropsied 6 h, 24 h, 48 h, 72 h, 7 days, 10 days, and 14 days post-LNP administration; livers were harvested at the time of necropsy.

For newborn studies, *Ugt1* KO mice and heterozygous and wild-type littermates received an i.v. injection with 0.1, 0.2, or 0.5 mg/kg LNP-encapsulated *hUGT1A1* mRNA or vehicle (20 mM Tris, 8% sucrose buffer [pH 7.4]) within 24 h of birth via the temporal vein. Mice were monitored for survival and serum total bilirubin levels throughout the in-life phase of the study.

Serum analyses

Blood was collected in serum separator tubes (Micro sample tube Serum Gel, 1.1 mL, screw cap, Sarstedt, Germany) and allowed to clot. Serum was isolated and sent to Antech Diagnostics (Irvine, CA, USA) to analyze total bilirubin levels.

Histological evaluation

Hematoxylin and eosin staining for histopathology and ISH were performed as described previously.[12] An experienced board-certified veterinary pathologist microscopically examined liver sections for histopathology. The severity of the tissue lesions was graded as follows: grade 1 (1+): minimal, an inconspicuous to barely noticeable histopathological feature (involves ~10% of the tissue in an average high-power field); grade 2 (2+): mild, a noticeable but not prominent histopathological feature (involves 10% to 25% average high-power field); grade 3 (3+): moderate, a prominent but not dominant histopathological feature (involves 25% to 50% of the tissue in an average

high-power field); grade 4 (4+): marked, a dominant but not overwhelming histopathological feature (involves 50% to 95% of the tissue in an average high-power field); and grade 5 (5+): severe, an overwhelming histopathological feature (involves greater than ~95% of the tissue in an average high-power field).

ISH images were quantified using ImageJ.[39] Four to five images per sample were acquired with a Leica DMi8 microscope using rhodamine and 4′,6-diamidino-2-phenylindole (DAPI) channels to visualize ISH signals (h*UGT1A1* mRNA) and nuclei, respectively. Images were converted to 8 bits, and the area of ISH staining and number of nuclei were determined after thresholding. The ratio of ISH-positive area per number of nuclei was determined for each image and averaged for each group.

Protein analysis by Western blot

Liver samples were snap frozen at the time of necropsy, and tissue homogenates were prepared in the cell lysis buffer (Thermo Fisher Scientific) containing protease inhibitor cocktails (Sigma, St. Louis, MO, USA) followed by sonication to shear DNA. These extracts (30 μg protein) were subjected to immunoblotting with human recombinant anti-UGT1A1 (Abcam, ab170858) and anti-β-actin (Abcam, ab20272) antibodies. The signal was developed with SuperSignal West Pico PLUS Chemiluminescent Substrate (Thermo Fisher Scientific), and images were acquired on the Bio-Rad ChemiDoc Imaging system. Densitometry was performed against β-actin using Bio-Rad ImageLab 6.1 following the manufacturer's guidelines. Data are presented as fold change relative to signal strength at 6 h.

Statistical analysis

Evaluation of the statistical differences in serum total bilirubin levels over time was performed by linear mixed-effect modeling. Comparisons of differences between all serum total bilirubin values between groups were performed using t tests. All values are presented as mean ± standard deviation (SD). A p value of <0.05 was considered significant.

DATA AVAILABILITY

All data discussed in the manuscript are available in the main text or the supplemental information.

SUPPLEMENTAL INFORMATION

Supplemental information can be found online at https://doi.org/10.1016/j.omtm.2023.02.007.

ACKNOWLEDGMENTS

We would like to thank the Program for Comparative Medicine and the Histopathology Core at the Gene Therapy Program. We would like to thank Christine Draper, Katherine Trostle, Peter Hewins, Brett Brown, and Kimberly Tulish for invaluable technical assistance. In addition, we would like to thank Hanying Yan for help with statistical analysis and Aditya Dandekar for their continued support of this project. This work was supported by internal funds from the University of Pennsylvania's Gene Therapy Program; the LNPs were provided by Moderna Inc.

AUTHOR CONTRIBUTIONS

Conceptualization, J.A.G., K.C., P.G.V.M., and J.M.W.; investigation, J.A.G., J.K.C., V.C., M.K.S., M.J., J.C.T., and E.L.B.; methodology, J.A.G.; supervision, J.A.G. and J.M.W.; writing – original draft, J.A.G. and M.J.; writing – review & editing, J.A.G., V.C., K.C., and J.M.W.; formal analysis, J.C.T. and E.L.B.; funding acquisition, J.M.W.

DECLARATION OF INTERESTS

J.M.W. is a paid advisor to and holds equity in iECURE, Scout Bio, Passage Bio, and the Center for Breakthrough Medicines (CBM). He also holds equity in the G2 Bio-associated asset companies. He has sponsored research agreements with Amicus Therapeutics, CBM, Elaaj Bio, FA212, G2 Bio, G2 Bio-associated asset companies, iECURE, Passage Bio, and Scout Bio, which are licensees of Penn technology. J.M.W. and J.A.G. are inventors on patents that have been licensed to various biopharmaceutical companies and for which they may receive payments. K.C. and P.G.V.M. are employees of Moderna, Inc., and hold equities from the company.

REFERENCES

1. Crigler, J.F., Jr., and Najjar, V.A. (1952). Congenital familial nonhemolytic jaundice with kernicterus. Pediatrics *10*, 169–180.
2. Crigler, J.F., Jr., and Najjar, V.A. (1952). Congenital familial nonhemolytic jaundice with kernicterus; a new clinical entity. AMA. Am. J. Dis. Child. *83*, 259–260.
3. Ritter, J.K., Crawford, J.M., and Owens, I.S. (1991). Cloning of two human liver bilirubin UDP-glucuronosyltransferase cDNAs with expression in COS-1 cells. J. Biol. Chem. *266*, 1043–1047.
4. Bosma, P.J., Chowdhury, N.R., Goldhoorn, B.G., Hofker, M.H., Oude Elferink, R.P., Jansen, P.L., and Chowdhury, J.R. (1992). Sequence of exons and the flanking regions of human bilirubin-UDP-glucuronosyltransferase gene complex and identification of a genetic mutation in a patient with Crigler-Najjar syndrome, type I. Hepatology *15*, 941–947. https://doi.org/10.1002/hep.1840150531.
5. Ritter, J.K., Yeatman, M.T., Ferreira, P., and Owens, I.S. (1992). Identification of a genetic alteration in the code for bilirubin UDP-glucuronosyltransferase in the UGT1 gene complex of a Crigler-Najjar type I patient. J. Clin. Invest. *90*, 150–155. https://doi.org/10.1172/JCI115829.
6. Arias, I.M., Gartner, L.M., Cohen, M., Ezzer, J.B., and Levi, A.J. (1969). Chronic nonhemolytic unconjugated hyperbilirubinemia with glucuronyl transferase deficiency. Clinical, biochemical, pharmacologic and genetic evidence for heterogeneity. Am. J. Med. *47*, 395–409.
7. Bosma, P.J., Seppen, J., Goldhoorn, B., Bakker, C., Oude Elferink, R.P., Chowdhury, J.R., Chowdhury, N.R., and Jansen, P.L. (1994). Bilirubin UDP-glucuronosyltransferase 1 is the only relevant bilirubin glucuronidating isoform in man. J. Biol. Chem. *269*, 17960–17964.
8. Strauss, K.A., Robinson, D.L., Vreman, H.J., Puffenberger, E.G., Hart, G., and Morton, D.H. (2006). Management of hyperbilirubinemia and prevention of kernicterus in 20 patients with Crigler-Najjar disease. Eur. J. Pediatr. *165*, 306–319. https://doi.org/10.1007/s00431-005-0055-2.
9. Strauss, K.A., Ahlfors, C.E., Soltys, K., Mazareigos, G.V., Young, M., Bowser, L.E., Fox, M.D., Squires, J.E., McKiernan, P., Brigatti, K.W., et al. (2020). Crigler-najjar syndrome type 1: pathophysiology, natural history, and therapeutic frontier. Hepatology *71*, 1923–1939. https://doi.org/10.1002/hep.30959.
10. Greig, J.A., Calcedo, R., Kuri-Cervantes, L., Nordin, J.M.L., Albrecht, J., Bote, E., Goode, T., Chroscinski, E.A., Bell, P., Richman, L.K., et al. (2018). AAV8 gene therapy for Crigler-Najjar syndrome in macaques elicited transgene T cell responses that are

resident to the liver. Mol. Ther. Methods Clin. Dev. *11*, 191–201. https://doi.org/10.1016/j.omtm.2018.10.012.

11. Greig, J.A., Nordin, J.M.L., Draper, C., Bell, P., and Wilson, J.M. (2018). AAV8 gene therapy rescues the newborn phenotype of a mouse model of Crigler-Najjar. Hum. Gene Ther. *29*, 763–770. https://doi.org/10.1089/hum.2017.185.
12. Greig, J.A., Nordin, J.M.L., Draper, C., McMenamin, D., Chroscinski, E.A., Bell, P., Gray, J.T., Richman, L.K., and Wilson, J.M. (2018). Determining the minimally effective dose of a clinical candidate AAV vector in a mouse model of Crigler-Najjar syndrome. Mol. Ther. Methods Clin. Dev. *10*, 237–244. https://doi.org/10.1016/j.omtm.2018.07.008.
13. Bočkor, L., Bortolussi, G., Iaconcig, A., Chiaruttini, G., Tiribelli, C., Giacca, M., Benvenuti, F., Zentilin, L., and Muro, A.F. (2017). Repeated AAV-mediated gene transfer by serotype switching enables long-lasting therapeutic levels of hUgt1a1 enzyme in a mouse model of Crigler-Najjar Syndrome Type I. Gene Ther. *24*, 649–660. https://doi.org/10.1038/gt.2017.75.
14. Bortolussi, G., Zentilin, L., Baj, G., Giraudi, P., Bellarosa, C., Giacca, M., Tiribelli, C., and Muro, A.F. (2012). Rescue of bilirubin-induced neonatal lethality in a mouse model of Crigler-Najjar syndrome type I by AAV9-mediated gene transfer. Faseb. J. *26*, 1052–1063. https://doi.org/10.1096/fj.11-195461.
15. Bortolussi, G., Zentillin, L., Vaníkova, J., Bockor, L., Bellarosa, C., Mancarella, A., Vianello, E., Tiribelli, C., Giacca, M., Vitek, L., and Muro, A.F. (2014). Life-long correction of hyperbilirubinemia with a neonatal liver-specific AAV-mediated gene transfer in a lethal mouse model of Crigler-Najjar Syndrome. Hum. Gene Ther. *25*, 844–855. https://doi.org/10.1089/hum.2013.233.
16. Collaud, F., Bortolussi, G., Guianvarc'h, L., Aronson, S.J., Bordet, T., Veron, P., Charles, S., Vidal, P., Sola, M.S., Rundwasser, S., et al. (2019). Preclinical development of an AAV8-hUGT1A1 vector for the treatment of Crigler-Najjar syndrome. Mol. Ther. Methods Clin. Dev. *12*, 157–174. https://doi.org/10.1016/j.omtm.2018.12.011.
17. Ronzitti, G., Bortolussi, G., van Dijk, R., Collaud, F., Charles, S., Leborgne, C., Vidal, P., Martin, S., Gjata, B., Sola, M.S., et al. (2016). A translationally optimized AAV-UGT1A1 vector drives safe and long-lasting correction of Crigler-Najjar syndrome. Mol. Ther. Methods Clin. Dev. *3*, 16049. https://doi.org/10.1038/mtm.2016.49.
18. Seppen, J., Bakker, C., de Jong, B., Kunne, C., van den Oever, K., Vandenberghe, K., de Waart, R., Twisk, J., and Bosma, P. (2006). Adeno-associated virus vector serotypes mediate sustained correction of bilirubin UDP glucuronosyltransferase deficiency in rats. Mol. Ther. *13*, 1085–1092. https://doi.org/10.1016/j.ymthe.2006.01.014.
19. Apgar, J.F., Tang, J.P., Singh, P., Balasubramanian, N., Burke, J., Hodges, M.R., Lasaro, M.A., Lin, L., Millard, B.L., Moore, K., et al. (2018). Quantitative systems pharmacology model of hUGT1A1-modRNA encoding for the UGT1A1 enzyme to treat Crigler-Najjar syndrome type 1. CPT Pharmacometrics Syst. Pharmacol. *7*, 404–412. https://doi.org/10.1002/psp4.12301.
20. Cullis, P.R., and Hope, M.J. (2017). Lipid nanoparticle systems for enabling gene therapies. Mol. Ther. *25*, 1467–1475. https://doi.org/10.1016/j.ymthe.2017.03.013.
21. Kauffman, K.J., Dorkin, J.R., Yang, J.H., Heartlein, M.W., DeRosa, F., Mir, F.F., Fenton, O.S., and Anderson, D.G. (2015). Optimization of lipid nanoparticle formulations for mRNA delivery in vivo with fractional factorial and definitive screening designs. Nano Lett. *15*, 7300–7306. https://doi.org/10.1021/acs.nanolett.5b02497.
22. Kauffman, K.J., Mir, F.F., Jhunjhunwala, S., Kaczmarek, J.C., Hurtado, J.E., Yang, J.H., Webber, M.J., Kowalski, P.S., Heartlein, M.W., DeRosa, F., and Anderson, D.G. (2016). Efficacy and immunogenicity of unmodified and pseudouridine-modified mRNA delivered systemically with lipid nanoparticles in vivo. Biomaterials *109*, 78–87. https://doi.org/10.1016/j.biomaterials.2016.09.006.
23. Pardi, N., Tuyishime, S., Muramatsu, H., Kariko, K., Mui, B.L., Tam, Y.K., Madden, T.D., Hope, M.J., and Weissman, D. (2015). Expression kinetics of nucleoside-modified mRNA delivered in lipid nanoparticles to mice by various routes. J. Contr. Release *217*, 345–351. https://doi.org/10.1016/j.jconrel.2015.08.007.
24. Pardi, N., Secreto, A.J., Shan, X., Debonera, F., Glover, J., Yi, Y., Muramatsu, H., Ni, H., Mui, B.L., Tam, Y.K., et al. (2017). Administration of nucleoside-modified mRNA encoding broadly neutralizing antibody protects humanized mice from HIV-1 challenge. Nat. Commun. *8*, 14630. https://doi.org/10.1038/ncomms14630.
25. Ramaswamy, S., Tonnu, N., Tachikawa, K., Limphong, P., Vega, J.B., Karmali, P.P., Chivukula, P., and Verma, I.M. (2017). Systemic delivery of factor IX messenger RNA for protein replacement therapy. Proc. Natl. Acad. Sci. USA *114*, E1941–E1950. https://doi.org/10.1073/pnas.1619653114.
26. Iyanagi, T., Watanabe, T., and Uchiyama, Y. (1989). The 3-methylcholanthrene-inducible UDP-glucuronosyltransferase deficiency in the hyperbilirubinemic rat (Gunn rat) is caused by a -1 frameshift mutation. J. Biol. Chem. *264*, 21302–21307.
27. Gunn, C.K. (1944). Hereditary acholuric jaundice in the rat. Can. Med. Assoc. J. *50*, 230–237.
28. Nguyen, N., Bonzo, J.A., Chen, S., Chouinard, S., Kelner, M.J., Hardiman, G., Bélanger, A., and Tukey, R.H. (2008). Disruption of the ugt1 locus in mice resembles human Crigler-Najjar type I disease. J. Biol. Chem. *283*, 7901–7911. https://doi.org/10.1074/jbc.M709244200.
29. Bortolussi, G., Baj, G., Vodret, S., Viviani, G., Bittolo, T., and Muro, A.F. (2014). Age-dependent pattern of cerebellar susceptibility to bilirubin neurotoxicity in vivo in mice. Dis. Model. Mech. *7*, 1057–1068. https://doi.org/10.1242/dmm.016535.
30. Seppen, J., van Til, N.P., van der Rijt, R., Hiralall, J.K., Kunne, C., and Elferink, R.P.J.O. (2006). Immune response to lentiviral bilirubin UDP-glucuronosyltransferase gene transfer in fetal and neonatal rats. Gene Ther. *13*, 672–677. https://doi.org/10.1038/sj.gt.3302681.
31. An, D., Frassetto, A., Jacquinet, E., Eybye, M., Milano, J., DeAntonis, C., Nguyen, V., Laureano, R., Milton, J., Sabnis, S., et al. (2019). Long-term efficacy and safety of mRNA therapy in two murine models of methylmalonic acidemia. EBioMedicine *45*, 519–528. https://doi.org/10.1016/j.ebiom.2019.07.003.
32. An, D., Schneller, J.L., Frassetto, A., Liang, S., Zhu, X., Park, J.S., Theisen, M., Hong, S.J., Zhou, J., Rajendran, R., et al. (2017). Systemic messenger RNA therapy as a treatment for methylmalonic acidemia. Cell Rep. *21*, 3548–3558. https://doi.org/10.1016/j.celrep.2017.11.081.
33. Jansen, P.L. (1999). Diagnosis and management of Crigler-Najjar syndrome. Eur. J. Pediatr. *158*, S89–S94.
34. Schauer, R., Stangl, M., Lang, T., Zimmermann, A., Chouker, A., Gerbes, A.L., Schildberg, F.W., and Rau, H.G. (2003). Treatment of Crigler-Najjar type 1 disease: relevance of early liver transplantation. J. Pediatr. Surg. *38*, 1227–1231. https://doi.org/10.1016/s0022-3468(03)00273-2.
35. van der Veere, C.N., Sinaasappel, M., McDonagh, A.F., Rosenthal, P., Labrune, P., Odièvre, M., Fevery, J., Otte, J.B., McClean, P., Bürk, G., et al. (1996). Current therapy for Crigler-Najjar syndrome type 1: report of a world registry. Hepatology *24*, 311–315. https://doi.org/10.1002/hep.510240205.
36. Fata, C.R., Gillis, L.A., and Pacheco, M.C. (2017). Liver fibrosis associated with Crigler-Najjar syndrome in a compound heterozygote: a case report. Pediatr. Dev. Pathol. *20*, 522–525. https://doi.org/10.1177/1093526617697059.
37. Nelson, J., Sorensen, E.W., Mintri, S., Rabideau, A.E., Zheng, W., Besin, G., Khatwani, N., Su, S.V., Miracco, E.J., Issa, W.J., et al. (2020). Impact of mRNA chemistry and manufacturing process on innate immune activation. Sci. Adv. *6*, eaaz6893. https://doi.org/10.1126/sciadv.aaz6893.
38. Hassett, K.J., Benenato, K.E., Jacquinet, E., Lee, A., Woods, A., Yuzhakov, O., Himansu, S., Deterling, J., Geilich, B.M., Ketova, T., et al. (2019). Optimization of lipid nanoparticles for intramuscular administration of mRNA vaccines. Mol. Ther. Nucleic Acids *15*, 1–11. https://doi.org/10.1016/j.omtn.2019.01.013.
39. Schneider, C.A., Rasband, W.S., and Eliceiri, K.W. (2012). NIH Image to ImageJ: 25 years of image analysis. Nat. Methods *9*, 671–675. https://doi.org/10.1038/nmeth.2089.

OMTM, Volume *29*

Supplemental information

Lipid nanoparticle-encapsulated mRNA therapy corrects serum total bilirubin level in Crigler-Najjar syndrome mouse model

Jenny A. Greig, Joanna K. Chorazeczewski, Vivek Chowdhary, Melanie K. Smith, Matthew Jennis, James C. Tarrant, Elizabeth L. Buza, Kimberly Coughlan, Paolo G.V. Martini, and James M. Wilson

Supplemental Data

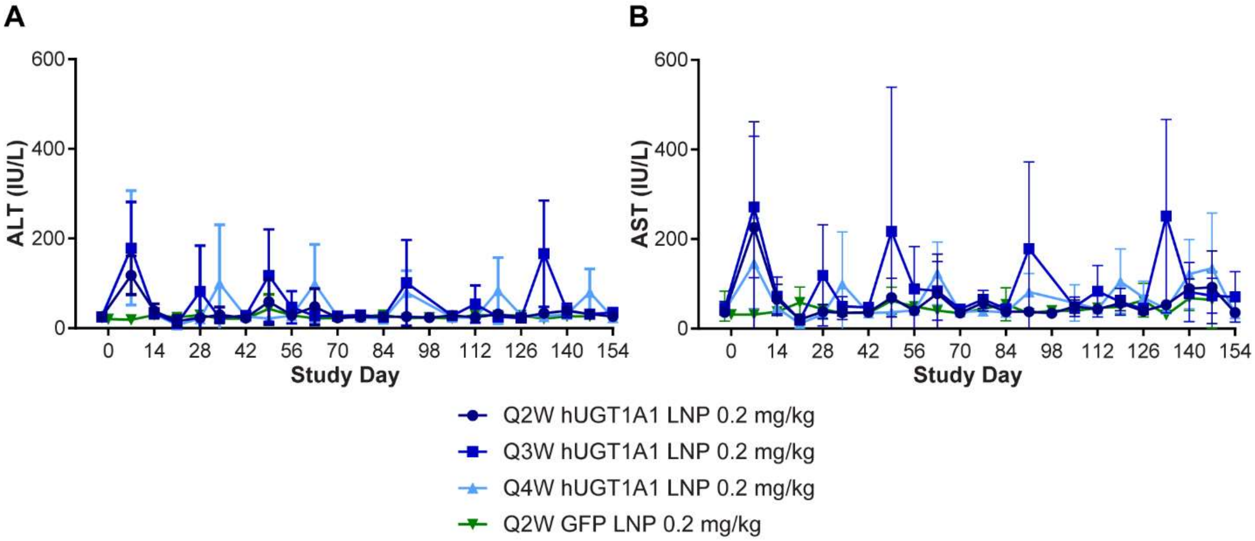

Figure S1. Liver transaminases following repeated administration of LNP encapsulated mRNA therapy in the *Ugt1* KO mouse.
Adult *Ugt1* KO mice (n=5/group) were injected IV with multiple administrations of 0.2 mg/kg LNP-encapsulated *hUGT1A1* mRNA (A) every two weeks (Q2W), (B) every three weeks (Q3W), and (D) every four weeks (Q4W). An additional group of mice received IV injections of 0.2 mg/kg LNP encapsulated GFP mRNA Q2W as a control. Blood was collected at selected time points to evaluate ALT (A) and AST (B). Values are presented as mean ± SD.

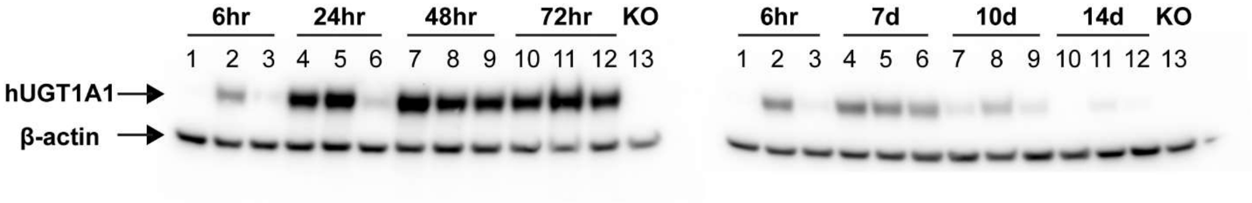

Figure S2. Evaluation of *hUGT1A1* protein pharmacokinetics following IV administration of LNP encapsulated *hUGT1A1* mRNA in C57BL/6J mice.
Adult C57BL/6J mice (n=3/group) were injected by IV with 0.5 mg/kg of LNP encapsulated *hUGT1A1* mRNA and sacrificed at the indicated time points. Livers were harvested for Western blot analysis to determine *hUGT1A1* protein expression.

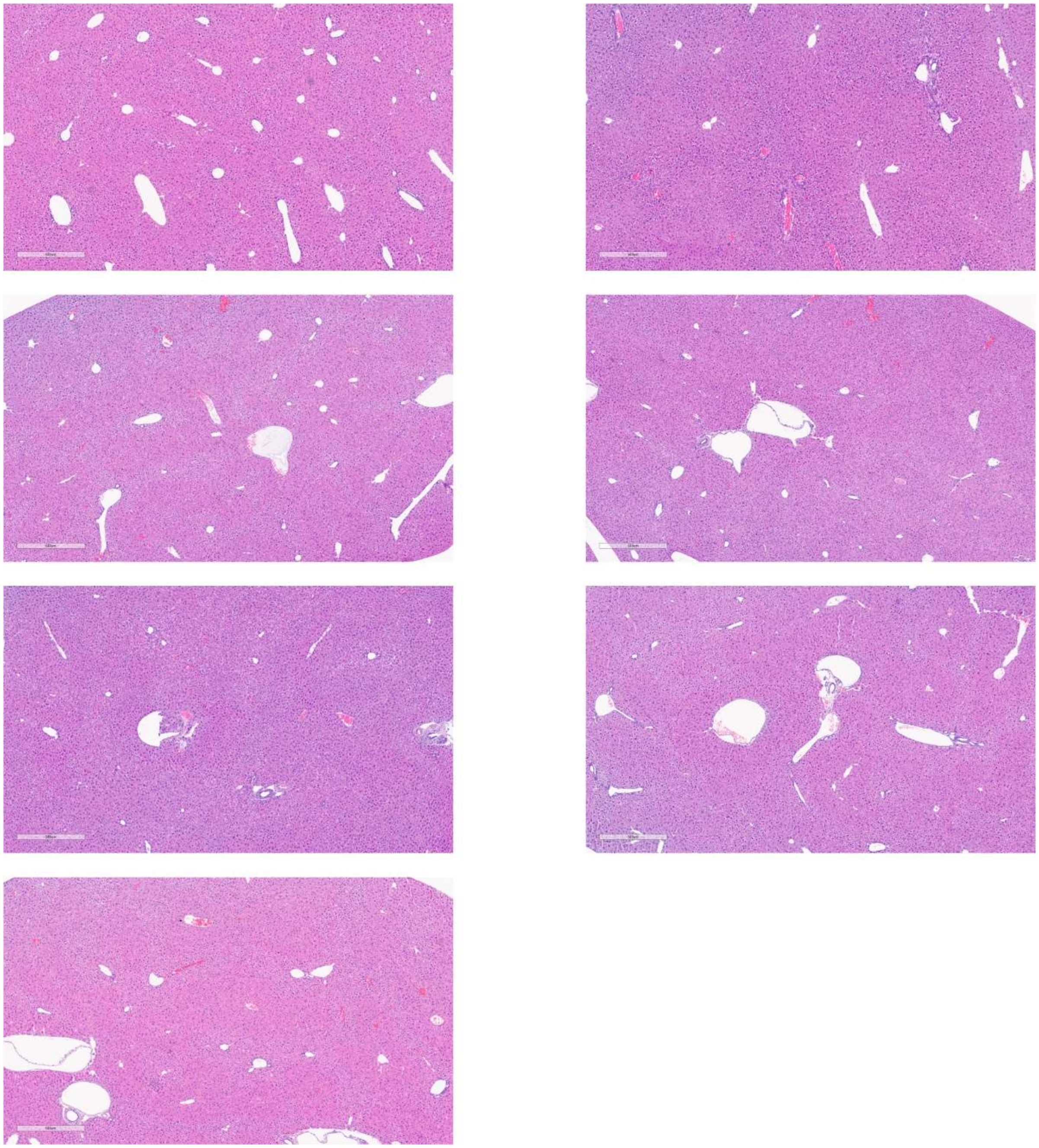

Figure S3. Images of hematoxylin and eosin (H&E)–stained liver sections of *Ugt1* KO mice. Newborn *Ugt1* KO mice were administered 0.5 mg/kg of LNP-encapsulated hUGT1A1 mRNA via a temporal vein, and tissues were harvested >100 days post-administration. Scale bar 500μm.
